# Identification of acquired tracheoesophageal fistula after tracheostomy decannulation by videofluoroscopic swallowing study: A case report

**DOI:** 10.1097/MD.0000000000025349

**Published:** 2021-04-02

**Authors:** Dong Ho Yoo, Min Soo Choi, Byeong Ju Lee, Yong Beom Shin, Jin A Yoon, Sang Hun Kim

**Affiliations:** a Department of Rehabilitation Medicine, Biomedical Research Institute, Pusan National University Hospital; b Department of Rehabilitation Medicine, Biomedical Research Institute, Pusan National University Hospital and Pusan National University School of Medicine, Busan, Republic of Korea.

**Keywords:** bronchoscopy, deglutition disorders, respiratory aspiration, tracheoesophageal fistula

## Abstract

**Rationale:**

Videofluoroscopic swallowing study (VFSS) is a noninvasive radiographic procedure that examines the oral, pharyngeal, and cervical esophageal stages of swallowing. Tracheoesophageal fistula (TEF) is difficult to diagnose depending on its size and location. However, how VFSS can be of benefit in the diagnosis of TEF has not been reported yet.

**Patient concerns:**

A 64-year-old man who had been tracheostomized post spinal tumor resection surgery at the cervical level 1 to 2, had his tracheostomy tube removed approximately 25 years ago. After decannulation, he reported coughing while swallowing food, foreign sensation in the neck and repeated bouts of pneumonia ever since.

**Diagnosis:**

VFSS revealed, for the first time, acquired TEF after tracheostomy decannulation as the cause of repetitive aspiration pneumonia.

**Intervention:**

VFSS was performed in this case.

**Outcomes:**

In the background of suspected TEF based on VFSS results, the patient underwent a computed tomography scan of the chest and airway in the prone position, followed by bronchoscopy, which confirmed the existence of a TEF. He then underwent primary closure of the fistula. The patient had an uneventful recovery and is currently symptom-free 10 months after the surgery.

**Lessons:**

This case alerts clinicians to the possibility of TEF as a diagnosis when the aspirate leaks from the upper esophagus and through the posterior wall of trachea in the esophageal phase of VFSS.

## 1 Introduction

Videofluoroscopic swallowing study (VFSS) is a noninvasive radiographic procedure that assesses the oral, pharyngeal, and cervical esophageal stages of swallowing.^[1]^ VFSS is widely used in the identification of deglutition disorders, particularly oropharyngeal deglutition disorders.^[2]^

The procedure of VFSS can be summarized as follows: the patient is seated upright and swallows barium-coated foods, including liquid, puree, semisolids, and solids in succession, which is recorded with dynamic radiographic imaging. With this procedure, the clinician can directly evaluate the swallowing function, including the biomechanical reasons for aspiration and the patient's compensation response. This can lead clinicians to identify interventions that can facilitate swallowing and increase its efficiency.

Tracheoesophageal fistula (TEF) is an abnormal pathway between the esophagus and trachea. Acquired TEF is a rare but difficult clinical issue. TEF can lead to continuous contamination of the respiratory system. Without proper treatment, mortality can occur from respiratory failure or malnutrition within 3 to 4 months.^[3]^

Acquired TEF, which occurs after tracheostomy, is a rare complication known to occur in less than 1% of tracheostomized patients.^[4]^ Insertion of the tracheostomy tube for prolonged periods can result in injury to the posterior tracheal wall, creating a communication between the esophagus and trachea.^[5]^ In this study, we report a case of acquired TEF, probably created by the tip of a tracheostomy tube, discovered for the first time by VFSS.

## 2 Case report

In 1992, a 36-year-old man complained of right hand numbness. He was diagnosed with a spinal tumor at the cervical 1 to 2 level on magnetic resonance imaging. Tracheostomy was performed after tumor resection in 1992, and decannulation was performed in 1994. Subsequently, the patient had difficulty swallowing food, foreign sensation in the neck, and coughing and used compensation methods such as lying down and pressing on his stomach forcefully to expel what was aspirated. He had suffered from repetitive pneumonia, but the reason of pneumonia had not been evaluated.

**Figure 1:**
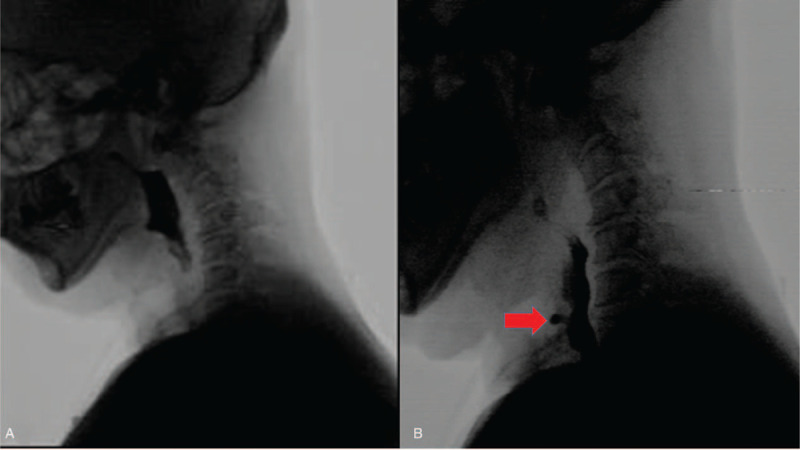
Videofluoroscopic swallowing study; swallowed barium liquid leaking from the upper esophagus (red arrow) to the posterior wall of trachea in the esophageal phase. (A) Before the swallowed barium passing through the TEF (B) After the swallowed barium passing through the TEF.

In 2020, the patient had a high fever of 39.3°C with dyspnea. He was admitted to our hospital for the first time and treated with antibiotics for pneumonia. To confirm the suspicion of aspiration pneumonia, VFSS was performed, which records the way patients swallow barium coated foods with dynamic radiographic imaging. On VFSS, liquid aspiration corresponding to a score of 7 on the Penetration-Aspiration Scale was confirmed.^[6]^ However, unlike common aspiration findings, the swallowed barium liquid leaked from the upper esophagus through the posterior wall of the trachea in the esophageal phase, which led us to suspect TEF (Fig. 1).

**Figure 2:**
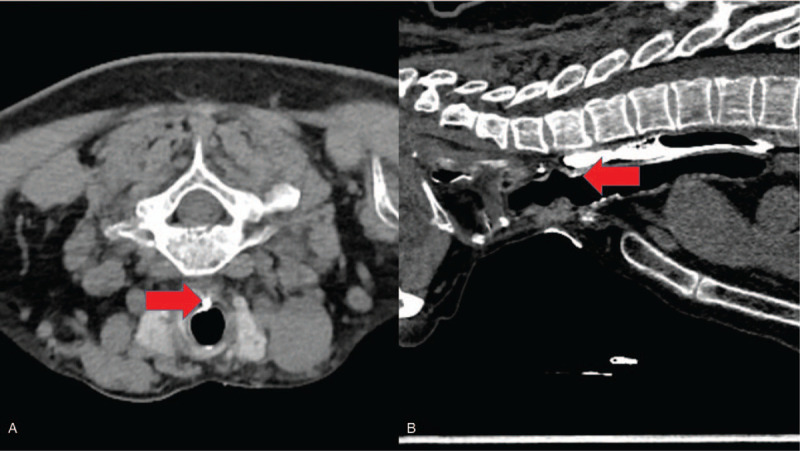
Computed tomography chest images in prone position presenting contrast leakage at the tracheoesophageal fistula (TEF) (red arrow) (A) Axial view (B) Sagittal view.

**Figure 3:**
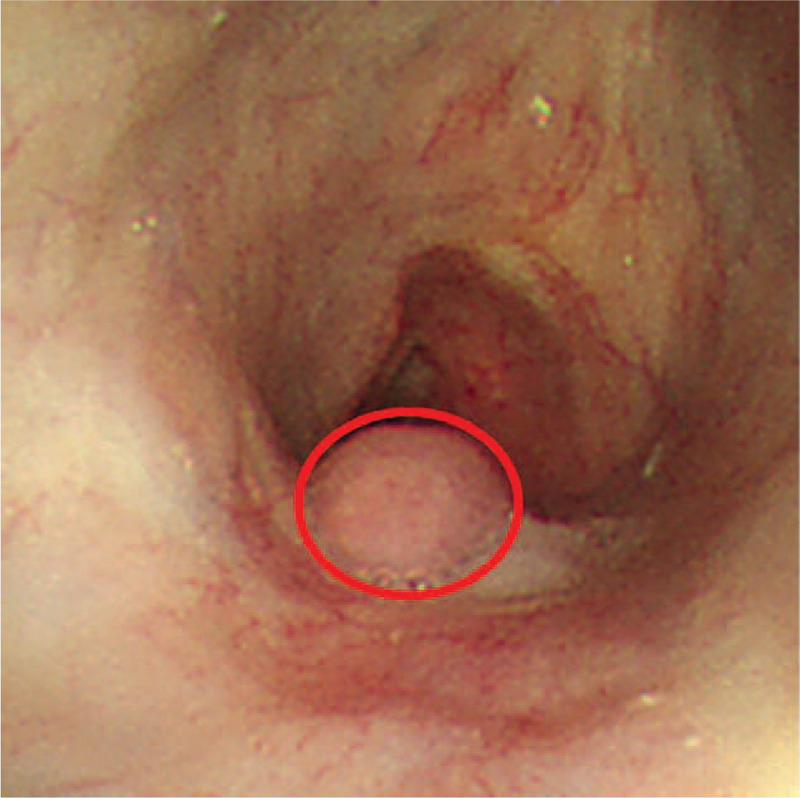
Bronchoscopy presenting a part of the esophagus, which looks like granulation tissue (red circle) on the posterior wall of the trachea, just below the vocal cords.

To confirm TEF, a computed tomography (CT) of the chest and airway in the supine position was performed, but no abnormal findings were observed. Because the contrast had passed into the posterior part of the esophagus by gravity, TEF, which was on the anterior part of the esophagus, could not be seen. The CT of the chest and airway was reexamined in the prone position, which is not typically done, to enable the swallowed contrast to slide into the airway under the influence of gravity. This protocol revealed the leakage of contrast into the trachea via TEF at the level of the thyroid gland (Fig. 2). Bronchoscopy revealed a part of the esophagus, which looked like granulation tissue, on the posterior wall of the trachea, just below the vocal cord (Fig. 3). Subsequently, the patient underwent lateral approach repair, which involves separation of trachea and esophagus, division of fistula and suture of esophageal and tracheal openings. The operation finding was that the fistula was located 2.2 cm below the cricoid cartilage and found to be about 6 mm in diameter. The patient had an uneventful recovery and is currently symptom-free 10 months after the surgery.

### 2.1 Ethical statement

Written consent was obtained from the patient for the publication of this case report. Our institutional review board does not require a formal ethical approval for case reports with written, informed consent.

## 3 Discussion

We report the case of a patient who had suffered from repetitive pneumonia for approximately 25 years after tracheostomy decannulation. The patient also had difficulty swallowing food, foreign sensation in the neck and cough, which are symptoms of aspiration. He used compensation methods such as lying down and pressing his own stomach forcefully to expel what was aspirated. However, this method could not completely prevent aspiration pneumonia. We successfully diagnosed acquired TEF in this patient with the help of VFSS, which has hitherto not been reported in the literature.

VFSS captures sequential videoradiographic images of barium-coated food and liquids as they pass through the oral, pharyngeal, and esophageal phases in real time.^[7]^ As VFSS helps visualize the flow of barium-coated food in real time, it is considered to be the preferred method for diagnosing deglutition disorders.^[8]^

In general, fiberoptic airway endoscopy and CT airway are typically performed for the diagnosis of TEF.^[9]^ Bronchoscopy aids visualization of the TEF directly, revealing both its location and extent. The exact site can be described with anatomical reference points, such as the vocal folds in a proximal fistula, and the tracheal carina in a distal fistula.^[10]^ TEF can also be diagnosed using a CT airway, which demonstrates the fistulous tract.^[10]^

Nonetheless, diagnosis of TEF can be rather difficult.^[11]^ Depending on its size and location, it is easy to miss even on bronchoscopy or CT airway. In our case, it could have been mistaken as a part of the esophagus pushed out through the fistula for granulation tissue. If the treating physicians had not suspected the possibility of a TEF, they would have had the granulation tissue which was actually a part of the esophagus removed, resulting medically dangerous conditions.

Generally, a CT airway is performed after the patient is administered an intravenous injection. However, a previous study has described how the process of swallowing can be determined by scanning patients in the supine position after they have swallowed the contrast medium.^[12]^ We used that protocol to determine TEF, but CT airway in the supine position did not present findings of TEF. Due to gravity pulling the contrast into the posterior part of the esophagus, it was not led into the TEF that originated from the anterior wall of the esophagus. A CT airway in the prone position, which is not typically done, was performed to make the contrast pass to the anterior part of the esophagus by gravity, which made the contrast slide into the TEF. Therefore, this test could show that TEF existed.

In conclusion, while VFSS is generally used to evaluate swallowing function, it can also be an intuitive and easy way to diagnose TEF, as in this case. Although it is difficult to determine TEF at the level of the thoracic esophagus, VFSS can be an effective method for diagnosing acquired TEF at the level of the cervical esophagus, which most commonly occurs as a complication of prolonged tracheal tube insertion. After detecting TEF with VFSS, other methods to diagnose TEF, such as CT airway in the prone position after the patients have swallowed the contrast medium, bronchoscopy, and esophagography should be performed sequentially to confirm TEF. Finally, acquired TEF can be initially identified using VFSS, which has sufficient diagnostic value, in patients suspected of having acquired TEF with recurrent pneumonia.

This study conforms to all CARE guidelines and reports the required information accordingly.^[13]^

## Acknowledgments

This research received no specific grant from any funding agency.

## Author contributions

**Conceptualization:** Min Soo Choi, Byeong Ju Lee, Sang Hun Kim.

**Data curation:** Dong Ho Yoo, Min Soo Choi, Jin A Yoon, Sang Hun Kim.

**Supervision:** Jin A Yoon, Yong Beom Shin, Sang Hun Kim.

**Visualization:** Dong Ho Yoo, Byeong Ju Lee.

**Writing – original draft:** Dong Ho Yoo.

**Writing – review & editing:** Sang Hun Kim.
